# Association of Built Environmental Features with Rates of Infectious Diseases in Remote Indigenous Communities in the Northern Territory, Australia

**DOI:** 10.3390/healthcare10010173

**Published:** 2022-01-17

**Authors:** Amal Chakraborty, Victor Maduabuchi Oguoma, Neil T. Coffee, Peter Markey, Alwin Chong, Margaret Cargo, Mark Daniel

**Affiliations:** 1 Research Centre for Palliative Care, Death and Dying, College of Nursing and Health Sciences, Flinders University, Bedford Park, SA 5042, Australia; 2 Health Research Institute, Faculty of Health, University of Canberra, Bruce, ACT 2601, Australia; victor.oguoma@canberra.edu.au (V.M.O.); neil.coffee@canberra.edu.au (N.T.C.); margaret.cargo@canberra.edu.au (M.C.); mark.daniel@canberra.edu.au (M.D.); 3 Centre for Disease Control, Top End Health Services, Northern Territory Department of Health, Northern Territory Government, Darwin, NT 0810, Australia; Peter.Markey@nt.gov.au; 4 Arney Chong Consulting, Adelaide, SA 5081, Australia; alwin.chong@arneychongconsulting.com; 5 South Australian Health and Medical Research Institute, Adelaide, SA 5000, Australia

**Keywords:** built environment, indigenous, communicable diseases, infectious diseases, disease outbreaks, community infrastructure, remote community, spatial epidemiology, public health

## Abstract

The health of Indigenous Australians is far poorer than non-Indigenous Australians, including an excess burden of infectious diseases. The health effect of built environmental (BE) features on Indigenous communities receives little attention. This study’s objective was to determine associations between BE features and infectious disease incidence rates in remote Indigenous communities in the Northern Territory (NT), Australia. Remote Indigenous communities (*n* = 110) were spatially joined to 93 Indigenous Locations (ILOC). Outcomes data were extracted (NT Notifiable Diseases System) and expressed as ILOC-specific incidence rates. Counts of buildings were extracted from community asset maps and grouped by function. Age-adjusted infectious disease rates were dichotomised, and bivariate binomial regression used to determine the relationships between BE variables and infectious disease. Infrastructure Shelter BE features were universally associated with significantly elevated disease outcomes (relative risk 1.67 to 2.03). Significant associations were observed for Services, Arena, Community, Childcare, Oval, and Sports and recreation BE features. BE groupings associated with disease outcomes were those with communal and/or social design intent or use. Comparable BE groupings without this intent or use did not associate with disease outcomes. While discouraging use of communal BE features during infectious disease outbreaks is a conceptually valid countermeasure, communal activities have additional health benefits themselves, and infectious disease transmission could instead be reduced through repairs to infrastructure, and more infrastructure. This is the first study to examine these associations simultaneously in more than a handful of remote Indigenous communities to illustrate community-level rather than aggregated population-level associations.

## 1. Introduction

Achieving and maintaining healthful living conditions is pivotal to reducing population risks for prevalent diseases, especially in disadvantaged communities [1]. A 2016 World Health Organisation (WHO) report estimated that some 22% of the global burden of disease, and 23% of all deaths, are attributable to modifiable environmental factors [2]. Disease burdens associated with environmental exposures are largely the result of non-communicable, chronic diseases such as cardiovascular disease, cancer, and chronic obstructive pulmonary disease, and communicable infectious and parasitic conditions such as lower respiratory infections, and diarrhea. The WHO is now calling for creating and maintaining healthful environments as a priority for primary prevention [2]. This call is supported by the Aboriginal and Torres Strait Islander (hereafter referred to as Indigenous) leadership in Australia who for decades have been advocating for improvements to living conditions in remote Australian communities [3,4,5]. Although the Australian federal government policy, Closing the Gap, has achieved some gains on maternal and child health, early childhood education, and year 12 educational attainment, substantial disparities in health status remain [6,7].

The health status of Indigenous Australians is poorer than non-Indigenous Australians [8], consistent with the health status of Indigenous peoples globally [9]. Estimated gaps in life expectancy at birth between Indigenous and non-Indigenous Australians are 10.6 years for males and 9.5 years for females, respectively [10]. For decades, a strong policy focus has targeted cardiometabolic disease inequities through attempts to improve individual-level health behaviours (e.g., smoking, diet, and insufficient physical activity) [11]—but not specifically the environments or contexts in which these behaviours occur.

Gaps in life expectancy, disease burden and hospitalisation experienced by Indigenous Australians are partially the result of preventable infectious and chronic diseases [12]. The characteristics of Indigenous socio-economic disadvantage, such as overcrowding and poor housing infrastructure including non-functional facilities for health and hygiene [13,14] promote infectious disease transmission. Consequently, infectious diseases burden the Indigenous population disproportionately, at a rate 11 times higher than in the non-Indigenous population, and with a 24-year difference in median age at death when infectious diseases are present [15]. Evidence suggests infectious pathogens can cause significant inflammation within the immune system resulting in the extensive sequelae and burden of non-communicable chronic diseases [16,17]. Therefore, recurrent bacterial and viral infections transmitted via environmental pathways are particularly threatening to long-term Indigenous health outcomes.

At the disease level, there are striking differentials in the incidence of invasive Group A Streptococcus (iGAS) infection between Indigenous and non-Indigenous Australian populations, with rates ranging between 32.2–82.5 per 100,000 persons and 6.4–10.2 per 100,000 persons, respectively [18]. Both invasive and non-invasive Group A streptococcal infections are precursors to both acute rheumatic fever and acute post-streptococcal glomerulonephritis (APSGN). Acute rheumatic fever can result in rheumatic heart disease (a potentially fatal condition) and frequent cases of APSGN contributes to chronic renal disease in the Indigenous population [19].

Australian Indigenous school-age children suffer the highest documented incidence of acute rheumatic fever in the world (194 per 100,000 individuals aged 5–14 years) [20], whereas this disease is almost non-existent in Australian born, non-Indigenous children [18]. Between 1997 and 2010, the incidence of acute rheumatic fever was 68.8 times higher in the Indigenous population (IRR; 95%CI = 40.1, 118.1) relative to the non-Indigenous population of the Northern Territory (NT) of Australia [20]. APSGN incidence in the NT between 1991 and July 2008 was 94.3 cases per 100,000 person-years in Indigenous children under 15 years of age, with the rate ratio 53.6 (95%CI = 32.6, 94.8) for Indigenous relative to non-Indigenous children [21].

Invasive pneumococcal disease (caused by *Streptococcus pneumoniae*) is a major cause of vaccine-preventable disease worldwide [22]. In vulnerable groups (typically infants, young children, and the elderly) the disease can spread from the nasopharynx to the respiratory tract, cerebrospinal, and/or pleural fluid, causing pneumonia, septicaemia, and meningitis [23]. Less acutely dangerous diseases, such as otitis media, can also arise subsequent to *Streptococcus pneumoniae* infection, causing hearing loss and contributing to poorer learning outcomes in school-aged children [24]. Nationally, invasive pneumococcal disease notification rates in Australia are disproportionately higher in the Indigenous relative to non-Indigenous population (Rate Ratio 6.5; 95%CI = 6.2, 6.9) [25].

Seasonal influenza has significantly higher notification and hospitalisation rates (RR 1.5 to 8.6, dependent upon age, and RR 1.2 to 4.3, dependent upon age, respectively) in Indigenous compared to non-Indigenous Australians [26]. The infection rate during the 2009 influenza pandemic (i.e., H1N1) was markedly higher in the Indigenous population (IRR 12; 95%CI = 7.8, 18.0) [27], than in the non-Indigenous population of the NT, as was the fatality rate (RR 3.35; 95%CI = 1.9, 5.5) [26]. Indigenous Australians with the H1N1 virus required hospitalisation at 3.2 to 3.4 times the rate of non-Indigenous Australians [27,28]. Evidence from Western Australia indicated that the presence of multiple co-morbidities was an independent predictor of hospitalisation from H1N1, whereas Indigenous status was not [28]. Thus, it appears that underlying elevated rates of chronic disease in the Australian Indigenous population, rather than any underlying innate genetic vulnerability [29], may have been responsible for the disproportionate effect of the H1N1 influenza pandemic on the Indigenous population. Given extensive evidence of continuing high rates of chronic conditions in the Indigenous population [30], it is likely that this vulnerability will continue to exacerbate the effect of disease outbreaks on these populations. This concern is magnified by the ongoing COVID-19 pandemic, which poses a substantial risk to the Indigenous populations, particularly in remote Indigenous communities, and requires the implementation of potentially culturally inappropriate countermeasures [31] such as social isolation. This measure may be challenging to enact given high levels of overcrowding and a tendency for extended family to live together.

In addition to recommendations to improve vaccination coverage and timeliness, non-vaccine related factors, including environmental factors, social disadvantage, and lack of access to culturally appropriate health services, have been raised as the most likely causes for continuing disease disparity between Indigenous and non-Indigenous Australians [25]. However, relatively little attention has been paid in the literature to the impact of the built environment (BE) on the prevalence of infectious diseases in the Indigenous Australian population. Most research attention on the BE has focused on Indigenous housing [32,33,34,35] which, while important, is only one BE feature that stands to influence Indigenous health. The field stands to benefit from greater consideration of non-housing related features of the BE. A substantial body of literature identifies associations between the BE and health in other settings [36]. This literature includes evidence relating urban BE features and morbidity rates for COVID-19 [37]. Poorer availability of non-housing related BE features has been recently shown to associate with higher rates of cardiometabolic disease-related morbidity and mortality in remote, predominantly Indigenous, communities in the NT [38]. For example, the presence of community-level BE features (e.g., women’s centres, and aged care facilities) has been linked to the spread of infectious diseases [39].

It is promising to see new evidence implicating non-housing related BE features associated with both communicable and non-communicable diseases in remote Indigenous communities in Northern Australia [39]. Addressing these broader environmental determinants has the potential to reduce health inequities experienced by the Indigenous population. However, this emphasis is under-researched and there remains a clear knowledge gap on the contribution to health of non-housing related BE features in remote Indigenous communities.

The purpose of this study was to evaluate for the years 2010–2015 the relationships between BE exposures and prevalent infectious diseases for remote, predominantly Indigenous communities in the NT of Australia.

## 2. Materials and Methods

### 2.1. Study Design and Ethics

This study was part of the Environments and Remote Indigenous Cardiometabolic Health (EnRICH) project, a cross-sectional, ecological epidemiological study using aggregated geographic and community-level data. Ethics approvals for this study were obtained from the Human Research Ethics Committee (HREC) of the University of South Australia (#31875, #33207), Central Australian HREC (#13-182), HREC of the NT Department of Health and Menzies School of Health Research (#2013-2083), and the University of Canberra HREC (blanket cross-institutional approval, 20 June 2017).

### 2.2. Setting

Communities were included within this study if they met the following eligibility criteria: (1) located within the borders of the NT of Australia, (2) population size 50 persons or more, (3) proportion of Indigenous residents 70% or more of the total community population, and (4) community defined as “remote” according to the Australian Standard Geographical Classification [40]. Eight hundred thirty-three Indigenous communities located within the NT were identified through the Australian Government Indigenous Programs and Policy Location (AGIL) 2013 dataset. Of these, 693 communities were excluded because their population was less than 50 persons, and 17 communities were excluded because the proportion of Indigenous Australians was less than 70%.

The resulting 123 AGIL-defined eligible communities were then matched to 104 Indigenous Locations (ILOCs), the smallest resolution at which census data for the Indigenous population are available from the Australian Bureau of Statistics (ABS) [40]. An ILOC typically represents a single Indigenous community, although for a small proportion of ILOCs the ABS aggregates small, geographically proximal communities to a single ILOC. Sociodemographic data were extracted from the ABS 2011 Population and Housing Census [41] and expressed at the level of the ILOC.

Of the 104 ILOCs, 13 contained more than one AGIL-defined community accounting for the “extra” 19 AGIL-based communities. Hence, 91 AGILs were a 1:1 match with an ILOC. The unit of observation and analysis was the ILOC. Where multiple communities were present within an ILOC, community-level outcome and BE exposure data were aggregated in the manner of the ABS, to create ILOC-level data.

### 2.3. Outcome Data

For each community, infectious disease data were obtained from the NT-wide Notifiable Diseases System, administered by the Centre for Disease Control (CDC), NT Department of Health. All such observations were assigned to a specific community via the common “resident location” record field. Five distinct sets of disease outcomes were compiled for each community for the period 1 January 2010 through 31 December 2015, extracted as counts and then analysed as incidence rates relative to ILOC-specific population denominators extracted from the ABS 2011 Census [41]. Outcomes of interest were rates of: (1) Gastrointestinal disease (*campylobacteriosis, cryptosporidiosis, salmonellosis* and *shigellosis* combined); (2) Group A streptococcal infection and sequelae (“Streptococcus”, *invasive Group A streptococcus, acute post-streptococcal glomerulonephritis,* and *acute rheumatic fever* combined); (3) laboratory-confirmed influenza; (4) invasive pneumococcal disease (*Streptococcus pneumoniae*); (5) total respiratory disease (*influenza* and *invasive pneumococcal disease* combined).

As a result of incomplete data on the five infectious disease outcomes being assessed, an additional 11 ILOCs were excluded, resulting in a sample of 93 ILOCs and 110 AGIL-defined communities.

### 2.4. Exposures Data

Access to core essential services and a healthful living environment are basic human needs for remote-dwelling Indigenous residents of the NT. As indicators of access to essential services and infrastructure, counts of buildings and other infrastructure elements were extracted from Serviced Land Availability Programme (SLAP) maps maintained by the NT Department of Lands, Planning, and the Environment. SLAP maps have been used for decades by the NT government to measure progress towards desired environmental health standards [42]. Here, as a basis for representing the community BE, SLAP maps were used to identify and determine the primary use of each building within a community, along with other community-level resources including sports fields and other infrastructure [38,43]. Features of the BE for each building or other infrastructure element were categorised according to function, purpose, or status, similar to an earlier epidemiological study [38]. BE features were dichotomised using either a median split (where multiple buildings within the same category existed) or based on their presence or absence. A measure of geographic isolation (“Isolation”, the presence or absence of other AGIL communities accessible within 300 km along the mainland road network) was also calculated.

### 2.5. Statistical Analysis

Descriptive analysis was conducted to summarise BE and outcome variables. Where appropriate, median and interquartile range (IQR) were used to describe continuous variables. Categorical variables were summarised as counts and percentages.

Age-adjusted rates of infectious diseases were dichotomised at their median values and a bivariate binomial regression with a logarithmic link function used to determine the relationship between BE variables (and Isolation) and infectious diseases outcomes.

Effect estimates are reported as relative risk (RR) with the 95% confidence interval (CI). Statistical significance was set at α = 0.05. Stata 16.1 (StataCorp, College Station, TX, USA) was used to conduct all statistical analysis.

## 3. Results

Characteristics of the sampled ILOCs and brief description of the BE features are presented in Table 1. The median population size was 222 (25th–75th percentile, 142–448), median age was 24 years (25th–75th percentile 21–26), and most mainland communities had another AGIL community accessible within 300 km (75 of 81, 92.6%). Most ILOCs lacked (or had low numbers of) features dedicated to Accommodation (available in 35.5% of ILOCs), Aged care (11.8%), Childcare (15.1%), Infrastructure transport (11.8%), Services (34.4%), Storage (18.3%), and Sports and recreation (37.6%). Total numbers of notified disease instances across the 6-year sample period were 1373 for gastrointestinal disease, 676 for Group A streptococcal infection and sequelae, 1171 for laboratory-confirmed influenza, 178 for invasive pneumococcal disease, and 1349 for total respiratory disease. The median rate (per 100 population over 6 years) of each disease was 2.6 (25th–75th percentile: 1.3–5.5) for gastrointestinal disease, 1.5 (25th–75th percentile: 0.8–2.7) for Group A streptococcal infection and sequelae, 2.1 (25th–75th percentile: 0.9–3.9) for laboratory-confirmed influenza, 0.2 (25th–75th percentile: 0.0–0.9) for invasive pneumococcal disease, and 2.8 (25th–75th percentile: 1.1–4.6) for total respiratory disease.

Bivariate relationships between age-adjusted rates of infectious diseases and BE features are presented in Table 2. A positive association between BE features and age-adjusted infectious disease rates was consistent across BE features including Infrastructure shelter, Community (e.g., community hall), Childcare, Services (e.g., Laundry), Sports and recreation, and Arena and Oval, although the strength of associations varied according to infectious disease.

The presence of Infrastructure shelter BE features was strongly associated with a greater risk of gastrointestinal disease (RR = 1.67; 95%CI = 1.07, 2.60), Group A streptococcal infection and sequalae (RR = 1.67; 95%CI = 1.07, 2.60), laboratory confirmed influenza (RR = 1.83; 95%CI = 1.16, 2.90), invasive pneumococcal disease (RR = 2.03; 95%CI = 1.26, 3.26), and total respiratory disease (RR = 1.83; 95%CI = 1.16, 2.90).

The presence of Community BE features was strongly associated with an elevated risk of gastrointestinal disease (RR = 1.93; 95%CI = 1.16, 3.20) and Group A streptococcal infection and sequelae (RR = 1.73; 95%CI = 1.06, 2.81). Childcare BE features was strongly associated with a greater risk of gastrointestinal disease and invasive pneumococcal disease (RR = 1.93; 95%CI = 1.40, 2.68) at the same magnitude.

Service and Arena BE features were strongly associated with a greater risk of Group A streptococcal infection and sequelae (RR = 1.54; 95%CI = 1.05, 2.26 vs. RR = 1.80; 95%CI = 1.06, 3.04), laboratory-confirmed influenza (RR = 1.68; 95%CI = 1.15, 2.46 vs. RR = 2.32; 95%CI = 1.29, 4.19), invasive pneumococcal disease (RR = 1.83; 95%CI = 1.25, 2.67 vs. RR = 2.03; 95%CI = 1.17, 3.54), and total respiratory disease (RR = 1.54; 95%CI = 1.05, 2.26 vs. RR = 2.68; 95%CI = 1.43, 5.04).

Additionally, Sports and recreation was strongly associated with an elevated risk of Group A streptococcal infection and sequelae and laboratory-confirmed influenza (RR = 1.59; 95%CI = 1.08, 2.34), at the same magnitude. Oval was positively associated with laboratory-confirmed influenza (RR = 2.11; 95%CI = 1.18, 3.79) and total respiratory disease (RR = 1.85; 95%CI = 1.07, 3.21). Other BE features such as Infrastructure transport (e.g., airstrip), Industry (e.g., fuel depot), Retail (e.g., community store), and Religion (e.g., church) were positively related to rates of infectious diseases, but the strength of such associations was weak.

## 4. Discussion and Conclusions

This ecological, community-level study describes associations between BE features and infectious disease rates in remote, predominantly Indigenous communities in the NT of Australia across the period 2010–2015. Frequent elevations (i.e., in 83.3% of features) in RR were observed for all BE feature–disease rate outcome associations, with only 16.6% of RR’s less than 1. This is the first study to examine these associations simultaneously in more than a handful of remote Indigenous communities to illustrate community-level rather than aggregated population-level associations.

A key finding was the association between disease outcomes and BE features having a communal purpose. Such locally accessible infrastructures create space for people to socially interact. For example, “Infrastructure shelter” was consistently associated with disease outcomes, whereas “Infrastructure transport” was not. The difference between these two categories of BE features is that “Infrastructure shelter” consists almost exclusively of “shade shelters”, facilities created to lessen the harsh environmental conditions of the NT and act as communal meeting places [44]. In contrast, “Infrastructure transport” features are exclusively bus shelters. Although these facilities provide shade and are used communally, they do not have the purpose of creating a social environment. As a result, both the exposure time and the number and variety of individual using these BE features is likely to be lower than where a social environment is created, thus limiting the opportunity for disease transmission.

Associations were observed between “Services” (predominantly law-enforcement related buildings) and most disease outcomes other than gastrointestinal disease). Outbreaks of infectious disease are rare in Australian prisons [45,46], but the capacity to minimise disease transmission via isolation or enforced separation in community-level police stations (the most common constituent of the “Services” category of BE features) is, arguably, far less than that available in other contexts (for example, health or education features), where quarantining procedures might be more readily adopted.

Associations between “Childcare” and “Community” buildings, which purposefully create social environments, and disease outcomes were observed in contrast to null or equivocal estimates for “Accommodation” and “Aged care” buildings, despite a range of factors that can predispose residents of aged care facilities to infection [47]. In both the “Accommodation” and “Aged care” categories, the social distancing of residents can be supported according to the design of the facilities: the degree of communality is more amenable to restriction than in “Community” or “Childcare” contexts which, by design, promote social interaction.

Associations were evident for “Sport and recreation” buildings (mainly recreation halls) and both Group A streptococcal infection and sequelae and laboratory-confirmed influenza. The similar “Arena” BE feature likewise associates with disease outcomes (apart from the null estimate for gastrointestinal disease). A dense concentration of individuals present at sporting events can enable infection transmission via the respiratory route (airborne and droplet) [48]. “Ovals”, however, afford a degree of separation for spectator crowds not possible within buildings, and thus a reduced frequency of associations with disease outcomes (when compared against the Arena category) in this study is unsurprising. Notably, all three “sports or physical activity” related BE features were associated with an elevated risk of laboratory-confirmed influenza transmission.

Our study reinforces the belief in some remote communities that life is often healthier away from built-up towns. These results could also naively support the perverse conclusion that banning gatherings in communal facilities or closing such important social facilities are reasonable preventive measures, despite benefits in other domains including mental and spiritual health. Yet, instead of discouraging gatherings, infectious disease transmission could be reduced through repairs to infrastructure, and more (or better) infrastructure. If, as in urban centres, communal infrastructure is required for day-to-day living, then it must coincide with hygiene education and account for risks associated with overcrowding. Infrastructure needs to be in good order, well maintained and sufficient to avoid or reduce risks related to overcrowding. This is because functional communal hygiene facilities and health hardware (e.g., safe electrical systems, toilets, showers, taps, kitchen cupboards and benches, and cooking and food preparation and storage facilities) fundamental to infectious disease prevention are prone to frequent and faster breakdown with high use [33]. Recent systematic reviews of studies assessing relationships between infectious diseases and housing featured housing condition, health hardware and overcrowding as the most common exposure variables examined [32,49]. These reviews found that crowding, conditions of dwelling characteristics and facilities, houses in need of repair/improvement, inadequate food preparation and storage areas, and poor sanitation and hygienic conditions were associated with gastrointestinal, respiratory, and streptococcal related infections [32,49]. Some 70% of the work required to improve these facilities is estimated due to inadequate maintenance and repairs [33].

Observations of BE features relating to elevated risk of infection are particularly relevant in the current context of the COVID-19 pandemic. COVID-19 shares transmission mechanisms with the infectious disease outcomes herein: contact, droplet [50] and transmission via fomites [51]. COVID-19 transmission at the origin of the pandemic (Wuhan City, China) [52] has been associated with BE features including building scale and retail sales area relative to total land area [37]. We draw no parallels between the BE of a major Chinese city and that of remote Indigenous Australian communities: the physical locations and built environments could hardly be more disparate. Rather, we highlight only that BE effects on infection rates are evident in both contexts and thus, given transmission mechanism similarities, it is conceivable that such effects could be present for COVID-19 in our sample. This is deeply concerning as the Indigenous population already carries a considerable burden for both chronic disease and, as seen with the H1N1 influenza pandemic of 2009, infectious disease too [27,28]. Any new COVID-19 outbreak is likely to interact with existing disease burdens to accelerate disproportionate increases in all-cause morbidity and mortality in remote Indigenous communities. Our findings further demonstrate the relevance of remote Indigenous community environmental features systematically identified and prioritised in our earlier scoping review [53] and concept mapping [54] studies. Such enquiry can support the environmental and public health sectors to select actionable priority BE indicators potentially preventing future infectious diseases outbreaks.

The effects of Isolation (i.e., presence of another community within 300 km) were varied, with small and large positive, but not statistically significant, associations. Despite the lack of statistical significance, gradations of remoteness appeared relevant to laboratory-confirmed influenza transmission, where less remote (i.e., not Isolated) communities were subject to substantially elevated risks of transmission.

### 4.1. Implications for Public Health

This study identified frequently elevated RRs for all infectious disease outcomes assessed across several categories of BE features. BE features thus appear to influence pathways of infectious disease transmission in these communities. This effect is most evident for those BE features designed or intended to encourage communality within the community—e.g., features such as Community buildings, Childcare facilities, and Infrastructure shelter features (shade shelters). Given the burden of infectious diseases on the Indigenous population, consideration should be given to controlling these transmission pathways, particularly where they are not immediately amenable to shut-down or spatial isolation enforcement (e.g., Services features, inclusive of law-enforcement related buildings, parts of which may be used for temporary incarceration).

In addition to the relevance of now commonly promulgated (under COVID-19) public health actions aimed at controlling, suppressing, or eliminating infectious disease outbreaks (social distancing, masks, and handwashing), healthful community features need to be recognised and supported with efforts made to create and maintain adequate infrastructure. Research shows that community capacity and features of built environment complement each other in their influence on collective health and wellbeing in remote Indigenous communities [53]. For example, while it is imperative to address inadequate quality housing (e.g., non-functional sanitation and hygiene facilities), it is also important to provide hygiene education and local opportunities for skill development and training in the maintenance, governance, and control of community health infrastructure.

Despite having been conducted in the NT of Australia, this study has potential relevance to remote-community dwelling Indigenous or First Nations populations, or any geographically clustered disadvantaged population, in other nations where the built environment could be improved to better support disease prevention.

### 4.2. Study Strengths

This study is the first, to our knowledge, to describe relationships between the availability of a broad range of individual building features on infectious disease rates in a comprehensive sample of remote Indigenous communities in the NT. These associations are described at the level of the ILOC, but ILOCs predominantly (82 of 93, 88.2%) represent a single community. As such, the associations we report are likely to hold at the community level also.

### 4.3. Limitations

This study has several limitations. We necessarily excluded 693 remote communities where population size was less than 50 persons. The disproportionate effect of very small numbers of cases within such small communities could have biased the reported associations. Excluded communities were unlikely to exhibit substantially developed built environments beyond individual residences, given small populations. Further, beyond the methodological rationale for exclusion and the likely limited BE in these communities, this study could not validly assess BE/infectious disease associations for all NT communities including the very large number of small Indigenous communities. As disease data were sourced from the NT Notifiable Diseases System, data are dependent on an individual’s ability to be tested. Remoteness of residential location can affect capacity to be tested and presents transport challenges for specimens sent from very isolated communities to a distant testing laboratory. These complicating factors may, in part, account for the lack of statistically significant associations between disease outcomes and Isolation.

We acknowledge as a source of bias the mobility of the Indigenous population [55,56], a flux that can cause variations in community population counts. Data regarding the number of people in the community at the time of the study are unlikely to be completely representative or exhaustive, and the “usual resident location” administrative designation might not be accurate for all residents. Such variation, however, is likely to be at random within the sample and, thus, would bias the results towards the null.

Regardless of these limitations, this study combines exceptional breadth of scope (110 communities within 93 ILOCs) in describing the relationship between the BE features of remote Indigenous communities and infectious disease rates, and thus attends to the knowledge gap relating to these factors in remote communities.

## Figures and Tables

**Table 1:** Characteristics of ILOCs including proportion of ILOCs with relevant BE features.

Built Environmental Features	Brief Description	Total (*n* = 93)
Infrastructure transport	Air/bus terminal, stops and shelters	11 (11.8%)
Infrastructure shelter	Shelters such as shade structures—parks, community settings, and ceremonies	50 (53.8%)
Accommodation	Motel/hotel and other commercial accommodation for visitors and tourists	33 (35.5%)
Community	Community services, council/community administration, art centres, women’s centres, and community centres	56 (60.2%)
Aged care	Housing and services for the aged population	11 (11.8%)
Childcare	Childcare and pre-school services	14 (15.1%)
Education	Primary/secondary schools, training centres and adult education	66 (71.0%)
Health	Health clinics and associated health services	64 (68.8%)
Services	Police, ranger, fire, Centrelink, housing, laundry, library services, and offices	32 (34.4%)
Industry	Buildings used for industry, workshops, service station, mechanic, plumber, and electrical	56 (60.2%)
Retail	Building used to sell goods, kiosk, and supermarket	52 (55.9%)
Religion	Church, convent, presbytery, or buildings used for religious services	44 (47.3%)
Sports and recreation	Offices, clubs, gyms, and halls for sporting and recreation	35 (37.6%)
Arena	Facilities for sports such as basketball/volleyball	60 (64.5%)
Oval	Sport field	62 (66.7%)
Storage	Sheds and storage buildings	17 (18.3%)
Unfinished building	Building under construction	57 (61.3%)
Disused	Unused/abandoned buildings	52 (55.9%)
Other features		
Isolation	Presence of another AGIL community within 300 km *	75 (92.6%)
Median age all persons	Median age of all persons in the community	24 (21–26)
Population size	Total number of persons in the community	222 (142–448)

Data are presented as *n* (%) for categorical measures and median (25th−75th percentile) for continuous measures. * mainland communities only (*n* = 81).

**Table 2:** Bivariate binomial regression of infectious disease outcomes on built environmental features and geographic isolation.

	Gastrointestinal Disease		Group A Streptococcal Infection and Sequelae		Laboratory-Confirmed Influenza		Invasive Pneumococcal Disease		Total Respiratory Disease	
BE Features and Isolation	RR (95%CI)	*p*	RR (95%CI)	*p*	RR (95%CI)	*p*	RR (95%CI)	*p*	RR (95%CI)	*p*
Infrastructure transport	1.09 (0.61, 1.95)	0.769	1.30 (0.79, 2.15)	0.296	1.53 (1.00, 2.34)	0.051	1.53 (1.00, 2.34)	0.051	1.53 (1.00, 2.34)	0.051
Infrastructure shelter	**1.67 (1.07, 2.60)**	**0.024**	**1.67 (1.07, 2.60)**	**0.024**	**1.83 (1.16, 2.90)**	**0.009**	**2.03 (1.26, 3.26)**	**0.003**	**1.83 (1.16, 2.90)**	**0.009**
Accommodation	0.77 (0.49, 1.22)	0.267	0.62 (0.38, 1.03)	0.064	0.94 (0.61, 1.44)	0.771	0.94 (0.61, 1.44)	0.771	0.85 (0.55, 1.33)	0.479
Community	**1.93 (1.16, 3.20)**	**0.011**	**1.73 (1.06, 2.81)**	**0.027**	1.56 (0.98, 2.49)	0.063	1.41 (0.90, 2.21)	0.136	1.41 (0.90, 2.21)	0.136
Aged care	0.51 (0.19, 1.36)	0.178	1.09 (0.61, 1.95)	0.769	1.53 (1.00, 2.34)	0.051	0.89 (0.45, 1.75)	0.731	1.53 (1.00, 2.34)	0.051
Childcare	**1.93 (1.40, 2.68)**	**<0.001**	1.34 (0.85, 2.10)	0.209	1.16 (0.70, 1.92)	0.571	**1.93 (1.40, 2.68)**	**<0.001**	1.16 (0.70, 1.92)	0.571
Education	1.07 (0.68, 1.69)	0.771	1.19 (0.74, 1.93)	0.470	1.19 (0.74, 1.93)	0.470	0.79 (0.53, 1.19)	0.260	1.07 (0.68, 1.69)	0.771
Health	1.07 (0.68, 1.67)	0.772	1.32 (0.81, 2.15)	0.262	1.19 (0.74, 1.89)	0.474	0.88 (0.58, 1.33)	0.538	1.07 (0.68, 1.67)	0.772
Services	1.41 (0.96, 2.08)	0.082	**1.54 (1.05, 2.26)**	**0.028**	**1.68 (1.15, 2.46)**	**0.008**	**1.83 (1.25, 2.67)**	**0.002**	**1.54 (1.05, 2.26)**	**0.028**
Industry	1.28 (0.83, 1.98)	0.269	1.56 (0.98, 2.49)	0.063	1.41 (0.90, 2.21)	0.136	1.06 (0.70, 1.61)	0.769	1.28 (0.83, 1.98)	0.269
Retail	1.27 (0.83, 1.94)	0.267	1.39 (0.90, 2.14)	0.134	1.39 (0.90, 2.14)	0.134	1.16 (0.77, 1.76)	0.478	1.16 (0.77, 1.76)	0.478
Religion	1.38 (0.92, 2.07)	0.121	1.50 (1.00, 2.27)	0.052	1.38 (0.92, 2.07)	0.121	1.38 (0.92, 2.07)	0.121	1.27 (0.85, 1.89)	0.252
Sports and recreation	1.46 (0.99, 2.15)	0.058	**1.59 (1.08, 2.34)**	**0.020**	**1.59 (1.08, 2.34)**	**0.02**	1.23 (0.82, 1.83)	0.313	1.46 (0.99, 2.15)	0.058
Arena	1.60 (0.97, 2.64)	0.064	**1.80 (1.06, 3.04)**	**0.028**	**2.32 (1.29, 4.19)**	**0.005**	**2.03 (1.17, 3.54)**	**0.012**	**2.68 (1.43, 5.04)**	**0.002**
Oval	1.64 (0.97, 2.75)	0.063	1.18 (0.75, 1.85)	0.477	**2.11 (1.18, 3.79)**	**0.012**	1.64 (0.97, 2.75)	0.063	**1.85 (1.07, 3.21)**	**0.028**
Storage	0.78 (0.43, 1.44)	0.428	0.92 (0.53, 1.59)	0.757	0.92 (0.53, 1.59)	0.757	0.92 (0.53, 1.59)	0.757	0.92 (0.53, 1.59)	0.757
Unfinished building	1.02 (0.67, 1.54)	0.934	0.72 (0.48, 1.06)	0.098	1.02 (0.67, 1.54)	0.934	0.93 (0.62, 1.40)	0.729	1.02 (0.67, 1.54)	0.934
Disuse	1.27 (0.83, 1.94)	0.267	1.27 (0.83, 1.94)	0.267	1.06 (0.71, 1.60)	0.764	1.53 (0.98, 2.38)	0.061	1.06 (0.71, 1.60)	0.764
Isolation (AGIL communities within 300 km) *	1		1.07 (0.47, 2.44)	0.879	3.20 (0.53, 19.39)	0.206	1.68 (0.53, 5.30)	0.376	3.28 (0.54, 19.86)	0.196

Note: Bold = significant at alpha = 0.05 AGIL: Australian Government Indigenous Policy and Program Location, BE: built environment, CI: confidence interval, and RR: relative risk. *—mainland communities only, *n* = 81.

## Data Availability

Not applicable.
